# Prognostic value of plasma NT-proBNP levels in very old patients with moderate renal insufficiency in China

**DOI:** 10.1007/s00391-017-1327-y

**Published:** 2017-10-20

**Authors:** Peng Gao, Qiwei Zhu, Suyan Bian, Hongwei Liu, Hongping Xie

**Affiliations:** 1 Department of Geriatric Cardiology, Chinese PLA General Hospital, 28 Fuxing Road, 100853 Beijing, Haidian District China; 2 Department of Rehabilitation and Physiotherapy, First Affiliated Hospital of PLA General Hospital, Beijing, China

**Keywords:** Prospective study, Aged, Chronic kidney disease, Risk factors, Cardiovascular disease, Prospektive Studie, Alter, Chronische Niereninsuffizienz, Risikofaktoren, Herz-Kreislauf-Erkrankung

## Abstract

**Background:**

The N‑terminal pro-brain natriuretic peptide (NT-proBNP) has an important prognostic value in chronic renal insufficiency; however, most studies have been conducted in patients with end-stage renal disease (ESRD). In this study we evaluated the prognostic significance of NT-proBNP in very old patients with stage 3 chronic kidney disease (CKD) and compared its prognostic value in CKD3a versus CKD3b patients.

**Methods:**

Patients (age ≥80 years old) hospitalized with stage 3 CKD from 2007 to 2010 who were eligible for this prospective study underwent follow-up examinations in 2015. The examinations included measurements of anthropometric characteristics, blood pressure, plasma NT-proBNP, creatinine, and lipids. End-point events were all-cause death and major adverse cardiac events (MACEs).

**Results:**

A total of 168 patients (mean age 87.4 ± 2.9 years, range 80–99 years) were included in the analysis (CKD3a, *n* = 117; CKD3b, *n* = 51). The results showed that CKD3b was associated with lower hemoglobin levels, higher NT-proBNP levels and a higher rate of hypertension compared with CKD3a. After a median follow-up of 3.8 years (interquartile range 1.5–6.1 years), a higher NT-proBNP level was associated with a higher risk of all-cause death (hazard ratio HR 1.986, 95% confidence interval CI 1.276–2.819, *p* = 0.028) and MACEs (HR 2.872, 95% CI 1.241–6.644, *p* = 0.014) after adjusting for age, sex, and traditional risk factors; however, a subgroup comparison showed that elevated NT-proBNP levels were associated with a higher risk of all-cause death (HR 2.350, 95% CI 1.906–6.091, *p* = 0.039) and MACEs (HR 3.025, 95% CI 1.024–8.940, *p* = 0.045) in CKD3a but not CKD3b.

**Conclusion:**

Levels of NT-proBNP increased with decreased renal function in very old patients with stage 3 CKD; therefore, NT-proBNP is an independent predictor for all-cause death and MACEs in these patients but has a greater prognostic value in CKD3a than in CKD3b.

## Zusammenfassung

**Hintergrund:**

Das „N-terminal pro-brain natriuretic peptide“ (NT-proBNP) hat einen wichtigen prognostischen Wert für die chronische Niereninsuffizienz; jedoch wurde die Mehrzahl der Studien bei Patienten mit einer Nierenkrankheit im Endstadium durchgeführt. In dieser Studie beurteilten wir die prognostische Bedeutung von NT-proBNP bei sehr alten Patienten mit chronischer Niereninsuffizienz („chronic kidney disease“, CKD) Stadium 3 und verglichen dessen prognostischen Wert bei CKD3a vs. CKD3b.

**Methoden:**

Patienten (Alter ≥80 Jahre), die von 2007 bis 2010 mit einer CKD Stadium 3 hospitalisiert wurden und die für diese prospektive Studie geeignet waren, unterzogen sich im Jahr 2015 Nachsorgeuntersuchungen. Die Untersuchungen umfassten Messungen anthropometrischer Werte, Blutdruck, NT-proBNP-Plasmakonzentration, Kreatinin und Lipide. Endpunktereignisse waren Tod jeglicher Ursache und schwere unerwünschte kardiale Ereignisse (MACEs).

**Ergebnisse:**

Insgesamt 168 Patienten (mittleres Alter: 87,4 ± 2,9 Jahre, Range: 80–99 Jahre) wurden in die Analyse eingeschlossen (CKD3a: *n* = 117; CKD3b: *n* = 51). Die Ergebnisse zeigten, dass CKD3b im Vergleich zu CKD3a mit geringeren Hämoglobinwerten, höheren NT-proBNP-Konzentrationen und einer höheren Hypertensionsrate assoziiert war. Nach einem medianen Follow-up von 3,8 Jahren (interquartile Spannweite: 1,5–6,1 Jahre) war eine höhere NT-proBNP-Konzentration mit einem erhöhten Risiko für Tod jeglicher Ursache (Hazard-Ratio [HR] 1,986; 95% Konfidenzintervall [CI] 1,276–2,819; *p* = 0,028) und MACEs (HR 2,872; 95% CI 1,241–6,644; *p* = 0,014) assoziiert, nachdem eine Anpassung für Alter, Geschlecht und traditionelle Risikofaktoren erfolgt war. Ein Subgruppenvergleich zeigte jedoch, dass eine erhöhte NT-proBNP-Konzentration in der CKD3a-Gruppe, jedoch nicht in der CKD3b-Gruppe, mit einem erhöhten Risiko für Tod jeglicher Ursache (HR 2,350, 95% CI 1,906–6,091; *p* = 0,039) und MACEs (HR 3,025, 95% CI 1,024–8,940; *p* = 0,045) assoziiert war.

**Schlussfolgerung:**

Die NT-proBNP-Konzentrationen stiegen mit abnehmender Nierenfunktion bei sehr alten Patienten mit CKD Stadium 3. Daher ist NT-proBNP ein unabhängiger Prädiktor für Tod jeglicher Ursache und MACEs bei dieser Patientengruppe, wobei es bei CKD3a einen größeren prognostischen Wert hat als bei CKD3b.

## Introduction

The number of old people is increasing along with the prevalence of chronic kidney disease (CKD) [1]. Zhang et al. [2] reported that the prevalence of CKD in the Chinese population was 10.8%, and Coresh et al. [3] found that the prevalence of CKD sharply rises with age, such that almost 50% of those older than 70 years are affected. Additionally, CKD is often associated with anemia, hypertension, and proteinuria, which increase the risk of cardiovascular disease, the primary cause of morbidity and premature mortality in CKD. Thus, old CKD patients have a higher risk of all-cause death or major adverse cardiac events (MACEs).

The N‑terminal pro-brain natriuretic peptide (NT-proBNP) is synthesized and secreted by the ventricular myocardium in response to volume or pressure overload or myocardial ischemia [4]. Previous studies have found that NT-proBNP levels are elevated in patients with CKD, and an elevated NT-proBNP level is an independent predictor of all-cause death and MACEs in all CKD stages [5, 6]; however, most studies evaluating the predictive value of NT-proBNP were conducted in patients with end-stage renal disease (ESRD) and on dialysis [5]. Patients with earlier stages of CKD also have a high rate of both fatal and nonfatal cardiovascular events [7]. Stage 3 CKD is classified as CKD3a if impairment of renal function is mild to moderate of CKD3b if impairment of renal function is moderate to severe. A decrease in renal function can be delayed and the risk of all-cause death and MACEs can be reduced if these patients receive comprehensive treatment; therefore, it is important to evaluate the risk of adverse events in patients with CKD stage 3. This study aimed to determine the prognostic value of NT-proBNP in very old (≥80 years old) patients with CKD stage 3 and compare the prognostic value of NT-proBNP in CKD3a versus CKD3b.

## Methods

### Study population

This prospective observational study included very old (age ≥80 years old) patients with stage 3 CKD who were hospitalized in the Department of Geriatric Internal Medicine in the People’s Liberation Army (PLA) General Hospital, Beijing, China. Patients with severe systemic diseases, such as collagenosis, inflammation, cachexia, severe liver disease, acute heart failure, or acute coronary syndrome, and those who had undergone coronary artery bypass grafting or percutaneous transluminal coronary angioplasty in the previous 6 months, were excluded. A total of 181 participants were eligible for the cross-sectional analysis from November 2007 to October 2010. Follow-up visits were conducted in April 2015. The median follow-up interval for the original 181 patients was 3.8 years (interquartile range IQR 1.5–6.1). Of the patients 13 were lost to follow-up and were therefore excluded from the final analysis. Complete follow-up data were obtained from 168 patients (follow-up rate 92.8%).

### Questionnaire and anthropometric measurements

Information about patient age, smoking status, and history of coronary heart disease (CHD), diabetes mellitus (DM), hypertension, atrial fibrillation (AF), and tumors was collected by the physician on admission to the hospital. The physical examination included measurement of height and weight. After the patient had been in a sitting position for ≥5 min, blood pressure was measured using a calibrated desktop sphygmomanometer (Yuyue, Armamentarium Limited Company, Jiangsu, People’s Republic of China), consistent with current recommendations. Blood pressure was measured three times consecutively with ≥1 min between measurements; the mean value was used for the statistical analysis.

Echocardiography was performed within 3 days after admission by an experienced echocardiography technician, who interpreted all scans. Left ventricular ejection fraction (LVEF) was determined using the biplane Simpson’s rule from apical 4‑chamber and 2‑chamber images of the heart [8]. Left atrial diameter (LAD), left ventricular end-systolic diameter (LVESD), left ventricular end-diastolic diameter (LVEDD), interventricular septum and posterior wall thickness (PWT) were measured on three consecutive heart beats and the results were averaged.

### Laboratory tests

All patients underwent a complete laboratory evaluation. Blood samples were collected from patients between 6 am and 8 am after overnight fasting (≥12 h). Peripheral blood samples were obtained to measure the following parameters: total cholesterol (TC), triglycerides, low-density lipoprotein cholesterol (LDL‑C), high-density lipoprotein cholesterol (HDL‑C), serum creatinine (sCr), blood urea nitrogen (BUN) and NT-proBNP. Concentrations of SCr were determined by an enzymatic assay (Roche Diagnostics, Basel, Switzerland) on a Hitachi 7600 autoanalyzer (Hitachi, Tokyo, Japan). The NT-proBNP level was determined with an electrochemiluminescence immunoassay (Roche Diagnostics, Mannheim, Germany) using a Roche analyzer (Roche Diagnostics, Indianapolis, IN).

### Definition of variables

We calculated the estimated glomerular filtration rate (eGFR) using the Chinese version of the modification of diet in renal disease equation as follows [9]: eGFR (ml/min/1.73 m^2^) = 175×standard creatinine (mg/dl)^−1.234^ × age (years)^−0.179^ × 0.79 (if female). The CKD was defined according to the clinical practice guidelines [10], and CKD3a and CKD3b were defined as eGFR 45–59 ml/min/1.73 m^2^ and 30–44ml/min/1.73 m^2^, respectively. Body mass index (BMI) was defined as weight (kg) divided by the square of the height (cm). We calculated left ventricular mass (LVM) as LVM = (0.8 [1.04 (LVEDd + PWTd + SWTd [interventricular septal end diastolic thickness])³ − (LVEDd)³]) + 0.6 g [8], body surface area (BSA) as BSA = 0.0061 × height + 0.0124 × weight^−0.0099^ [11], and left ventricular mass index (LVMI) as LVMI = LVM/BSA. Hypertension was defined as (i) systolic blood pressure ≥140 mm Hg, (ii) diastolic blood pressure ≥90 mm Hg and/or (iii) use of an antihypertensive drug. Diabetes mellitus was indicated by the use of a hypoglycemic drug or insulin [12]. The diagnoses of CHD, AF, and tumors were confirmed by medical history.

### Follow-up

Each patient medical record was reviewed by physicians every year of the baseline assessment. The major endpoints were all-cause death and MACEs. Death was ascertained from the death certificate, a legal document that includes time, place, and other information. The MACEs included nonfatal myocardial infarction, coronary revascularization therapy, and newly diagnosed coronary artery disease by coronary artery imaging or stroke.

### Statistical analysis

Continuous variables with a normal distribution are expressed as mean and standard deviation. Categorical variables are expressed as numbers and percentages. The NT-proBNP level is presented as a continuous variable (after natural logarithmic transformation). All analyses were performed at a median follow-up interval of 3.8 years (IQR 1.5–6.1). Continuous variables (demographic and clinical characteristics) were compared between groups by analysis of variance or Cuzick’s nonparametric test for trend. Proportions were compared using the χ^2^-test and Fisher’s exact test. The relationship between NT-proBNP levels and major end-points was evaluated using Cox proportional hazard regression model analysis. Model 1 was adjusted for age and sex, model 2 was adjusted for the variables in model 1 plus hypertension, DM, CHD, AF, tumor, lipid profile, hemoglobin (Hb), BMI, plasma albumin (ALB), mean systolic blood pressure, mean diastolic blood pressure, mean arterial pressure and pulse pressure, model 3 was adjusted for the variables in model 2 plus the use of major cardiovascular drugs, such as antiplatelet drugs, statins, renin-angiotensin system inhibitors, and beta-blockers and model 4 was adjusted for the variables in model 3 plus echocardiography parameters, such as LVEF, LAD, and LVMI. Differences in hazard ratios (HR) between patients with CKD3a and CKD3b were checked with interaction analysis. The cumulative incidences of mortality and MACEs were estimated using the Kaplan-Meier method. A receiver operating characteristic (ROC) curve was generated to evaluate the accuracy of NT-proBNP in the prediction of all-cause death and MACEs. All analyses were conducted using SPSS software for Windows, version 13.0 (SPSS, Chicago, IL); *p* values < 0.05 were considered significant.

## Results

### Patient characteristics

A total of 168 patients (mean age 87.4 ± 2.9 years, range 80–99) with moderate renal insufficiency were included in the analysis. There were 117 patients with CKD3a (mean age 87.4 ± 2.7 years) and 51 patients with CKD3b (mean age 87.7 ± 3.4 years). Demographic characteristics, cardiovascular risk factors, and related laboratory test results in each group are shown in Table 1. The two groups did not differ significantly with respect to the prevalence of CHD, DM, AF or tumors or use of antiplatelet drugs, statins and renin-angiotensin system inhibitors. Patients with CKD3b had a higher prevalence of hypertension (96.1% vs. 76.9%, *p* = 0.002), lower Hb levels (116 ± 15 g/l vs. 122 ± 13 g/l, *p* < 0.001), and higher plasma NT-proBNP levels (1227.7 ± 1177.8 pg/ml vs. 785.4 ± 752.0 pg/ml, *p* = 0.021) compared with patients with CKD3a. In addition, the use of beta-blockers was significantly higher in patients with CKD3b (51.0% vs. 34.2%, *p* = 0.041).

**Table 1:** Baseline characteristics of patients included in this study

Parameter	CKD3a	CKD3b	*p*-value
*n*	117	51	
Age (mean, years)	87.4 ± 2.7	87.7 ± 3.4	0.626
CHD (%)	95 (81.2%)	44 (86.2%)	0.426
HT (%)	90 (76.9%)	49 (96.1%)	0.002
AF (%)	20 (17.1%)	8 (15.7%)	0.823
DM (%)	59 (50.4%)	29 (56.9%)	0.446
Tumor (%)	23 (19.7%)	7 (13.7%)	0.359
NT-proBNP (pg/mL)	785.4 ± 752.0	1227.7 ± 1177.8	0.021
eGFR (ml/min/1.73 m^2^)	53.7 ± 4.0	38.1 ± 3.6	<0.001
SCr (µmol/l)	117.3 ± 9.1	157.3 ± 16.8	<0.001
UN (mmol/l)	9.0 ± 2.0	12.1 ± 3.7	<0.001
UA (µmol/l)	372.3 ± 79.6	385.8 ± 76.9	0.428
TC (mmol/l)	4.1 ± 0.6	4.1 ± 0.9	0.771
LDL‑C (mmol/l)	2.3 ± 0.5	2.3 ± 0.7	0.861
HDL‑C (mmol/l)	1.1 ± 0.2	1.1 ± 0.3	0.565
TG (mmol/l)	1.5 ± 0.7	1.6 ± 0.5	0.744
Hb (g/l)	122 ± 13	116 ± 15	*p* < 0.001
Plasma albumin (g/l)	39.3 ± 2.6	38.8 ± 2.5	0.38
BMI	24.0 ± 2.4	23.6 ± 2.5	0.449
mSBP (mm Hg)	129.6 ± 8.9	128.7 ± 9.0	0.636
mDBP (mm Hg)	67.7 ± 5.8	66.5 ± 5.7	0.334
MBP (mm Hg)	88.3 ± 6.0	87.2 ± 5.6	0.379
PP (mm Hg)	62.0 ± 8.3	62.2 ± 9.3	0.885
LVEF (%)	59.5 ± 4.0	59.1 ± 4.1	0.686
LAD (mm)	37.3 ± 3.2	37.6 ± 3.2	0.707
LVMI (g/m^2^)	127.7 ± 22.0	127.2 ± 24.8	0.918
Antiplatelet drugs (%)	80 (68.4%)	35 (68.6%)	0.974
Statin drugs (%)	50 (42.7%)	23 (45.1%)	0.778
CCB (%)	64 (54.7%)	29 (56.9%)	0.797
ACEI/ARB (%)	57 (48.7%)	27 (52.9%)	0.617
Beta-blocker (%)	40 (34.2%)	26 (51.0%)	0.041

*CHD* coronary heart disease, *HT* hypertension, *AF* atrial fibrillation, *DM* diabetes mellitus, *NT-proBNP* N-terminal pro-brain natriuretic peptide, *eGFR* estimated glomerular filtration rate, *SCr* serum creatinine, *UN* urea nitrogen, *UA* uric acid, *TC* total cholesterol, *LDL‑C* low-density lipoprotein cholesterol, *HDL‑C* high-density lipoprotein cholesterol, *TG* triglyceride, *Hb* hemoglobin, *BMI* body mass index, *mSBP* mean systolic blood pressure, *mDBP* mean diastolic blood pressure, *MBP* mean blood pressure, *PP* pulse pressure, *LVEF* left ventricular ejection fraction, *LAD* left atrial diameter, *LVMI* left ventricular mass index, *CCB* calcium channel blocker, *ACEI* angiotensin-converting enzyme inhibitor, *ARB* angiotensin receptor antagonist

### Prognostic value of NT-proBNP for all-cause death and MACEs in patients with stage 3 CKD

During follow-up 101 of the 168 patients (60.1%) died. Of these patients 16 (15.8%) died of cardiac causes, 39 (38.6%) died of infections and 26 (25.7%) died of progressive cancer. In addition, 67 patients (39.9%) suffered a MACE. Results of Cox proportional hazard model analysis showed that a higher NT-proBNP level was associated with a higher risk of all-cause death (HR 1.986, 95% confidence interval CI 1.276–2.819, *p* = 0.028) and MACEs (HR 2.872, 95% CI 1.241–6.644, *p* = 0.014) after adjusting for age, sex, BMI, comorbidities (CHD, hypertension, DM, and AF), lipid profiles, Hb, ALB, and major cardiovascular drugs (antiplatelet drugs, statins, calcium channel blockers, angiotensin-converting enzyme inhibitors, angiotensin receptor blockers, and beta-blockers); however, after further adjustment for LVEF, LAD, and LVMI (model 4), the NT-proBNP level was not associated with a higher risk of all-cause death and MACEs (Table 2).

**Table 2:** Association of baseline NT-proBNP level with all-cause mortality and MACEs

All-cause death	Model Cox	*p*-value	HR	95% CI		MACEs	*p*-value	HR	95% CI	
				Lower	Upper				Lower	Upper
*n* = 168										
CKD3	HR unadjusted	0.023	1.482	1.195	2.207		0.041	1.711	1.021	2.866
	Model 1	0.033	1.568	1.038	2.369		0.043	1.699	1.016	2.843
	Model 2	0.033	1.727	1.046	2.852		0.007	3.050	1.364	6.817
	Model 3	0.028	1.986	1.276	2.819		0.014	2.872	1.241	6.644
	Model 4	0.350	1.348	0.720	2.524		0.095	2.265	0.868	5.907
*n* = 117										
CKD3a	HR unadjusted	0.022	1.592	1.292	2.734		0.047	1.892	0.996	3.595
	Model 1	0.035	1.618	1.336	2.797		0.047	1.873	0.996	3.522
	Model 2	0.014	2.880	1.239	6.695		0.005	4.417	1.573	12.405
	Model 3	0.039	2.350	1.906	6.091		0.045	3.025	1.024	8.940
	Model 4	0.294	1.777	0.607	5.201		0.334	2.036	0.481	8.615
*n* = 51										
CKD3b	HR unadjusted	0.354	1.326	0.730	2.411		0.747	1.228	0.352	4.283
	Model 1	0.245	1.469	0.768	2.811		0.567	1.485	0.384	5.749
	Model 2	0.603	1.322	0.461	3.788		0.076	1.303	0.549	4.351
	Model 3	0.209	0.325	0.056	1.873		0.263	2.457	1.055	4.181
	Model 4	0.675	1.562	0.194	12.611		0.735	2.129	0.873	5.759

*HR* hazard ratio; *CI* confidence interval, *CKD* chronic kidney disease, *MACEs* major adverse cardiovascular events

*Model 1* adjusted for age and sex

*Model 2* adjusted for variables in model 1 plus the traditional cardiovascular risks CHD, hypertension, DM, AF, TC,TG, LDL‑C, HDL‑C, Hb, plasma albumin, BMI

*Model 3* adjusted for variables in model 2 plus therapeutic drugs, such as antiplatelet drugs, statins, CCB, ACEI/ARB, beta-blocker

*Model 4* adjusted for variables in model 3 plus LVEF, LAD and LVMI, MACEs

**Fig. 1:**
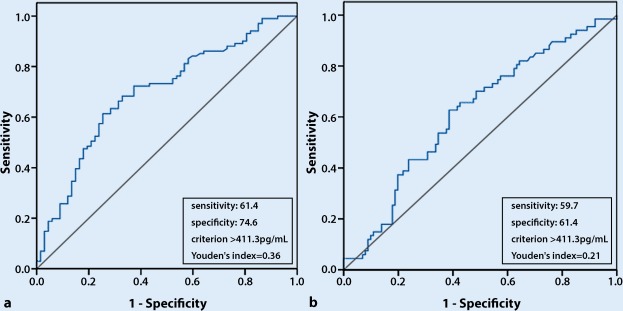
**a** An ROC curve of NT-proBNP to predict all-cause death in patients with CKD3. AUC was 0.697 (95% CI 0.616–0.778, *p* < 0.001). **b** An ROC curve of NT-proBNP to predict MACEs in patients with CKD3. The AUC was 0.616 (95% CI 0.530–0.701, *p* = 0.011). *ROC* receiver operating characteristic, *NT-proBNP* N-terminal pro-brain natriuretic peptide, *MACEs* major adverse cardiovascular events, *AUC* area under curves,* Cl* confidence interval. The *black* line is reference line. ​The *blue* line is ROC curve. The X-axes represents sensitivity, and the Y-axes represents specificity

Results of the ROC analysis indicated that NT-proBNP had a reasonable accuracy for predicting all-cause death and MACEs. The area under the ROC curve was 0.697 (95% CI 0.616–0.778, *p* < 0.001) for all-cause death (Fig. 1a). The cut-off NT-proBNP level for predicting all-cause death was 411.3 pg/ml and had a maximum Youden index of 0.360. For MACEs, the area under the ROC curve was 0.616 (95% CI 0.530–0.701, *p* = 0.011) (Fig. 1b), the cut-off NT-proBNP level for predicting MACEs was 411.3 pg/ml and the maximum Youden index was 0.241.

### Prognostic value of NT-proBNP levels in CKD3a versus CKD3b

**Fig. 2:**
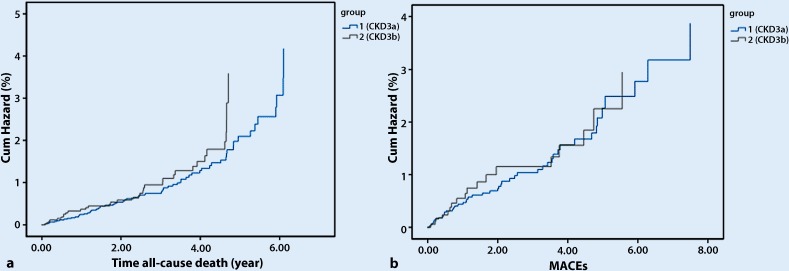
**a** Kaplan-Meier cumulative hazard of all-cause death in CKD3a and CKD3b. Incidence of all-cause death in CKD3b (70.6%) was significantly higher than in CKD3a (55.6%, log-rank test *p* = 0.037). **b** Kaplan-Meier cumulative hazard of MACEs in CKD3a and CKD3b. Incidence of MACEs in CKD3b (37.3%) was similar to CKD3a (41.0%). Log-rank test *p* = 0.563. The *black* line is reference line. ​The *blue* line is ROC curve. The X-axes represents sensitivity, and the Y-axes represents specificity

Assessment of endpoints during the follow-up period showed that in the CKD3a group, 65 patients (55.6%) died and 48 patients (41.0%) underwent MACEs. In the CKD3b group, 36 patients (70.6%) died and 19 (37.3%) suffered MACEs. Kaplan-Meier survival analysis showed that survival was lower in the CKD3b group (*p* = 0.037), but the incidence of MACEs did not differ significantly between the two subgroups (*p* = 0.563) (Fig. 2).

Results of Cox proportional hazard model analysis showed that in patients with CKD3a, a higher NT-proBNP level was associated with a higher risk of all-cause death (HR 2.350, 95% CI 1.906–6.091, *p* = 0.039) and MACEs (HR 3.025, 95% CI 1.024–8.940, *p* = 0.045) after adjusting for multiple risk factors (model 3); however, in patients with CKD3b, NT-proBNP level had no predictive value for all-cause death and MACEs after adjusting for multiple risk factors (model 3) (Table 2). By interaction analysis, the multiple adjusted HR values of NT-proBNP to predict all-cause death (2.350 vs. 0.325, *p* = 0.009) and MACEs (3.025 vs. 2.457, *p* = 0.012) were significantly higher in patients with CKD3a compared with CKD3b.

**Fig. 3:**
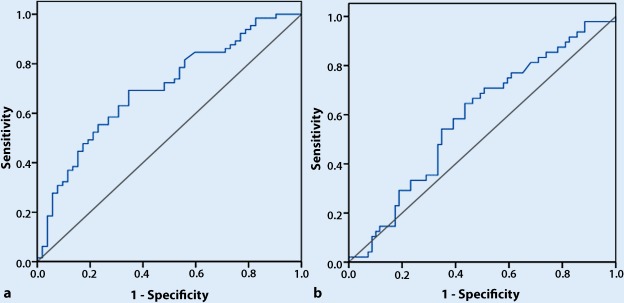
**a** An ROC curve of NT-proBNP to predict all-cause death in patients with CKD3a. AUC was 0.699 (95% CI 0.604–0.794, *p* < 0.001). **b** An ROC curve of NT-proBNP to predict MACEs in patients with CKD3a. AUC was 0.588 (95% CI 0.484–0.692, *p* = 0.106). *ROC* receiver operating characteristic, *NT-proBNP* N-terrninal pro-brain natriuretic peptide, *MACEs* major adverse cardiovascular events, *AUC* area under the curve, *Cl* confidence interval. *Blue line*: ROC curve; *black line*: reference line

In patients with CKD3a the ROC analysis indicated that NT-proBNP had a reasonable accuracy for predicting all-cause death but not for predicting MACEs. The area under the ROC curve was 0.699 (95% CI 0.604–0.794, *p* < 0.001) for all-cause death (Fig. 3a). The cut-off NT-proBNP level for predicting all-cause death was 288.9 pg/ml and had a maximum Youden index of 0.346. In patients with CKD3b, the area under the ROC curve was 0.588 (95% CI 0.484–0.692, *p* = 0.106) for MACEs (Fig. 3b). The ROC analysis indicated that NT-proBNP had no value for predicting all-cause death and MACEs in patients with CKD3b.

## Discussion

The main findings of this study include the following: first, in very old patients with stage 3 CKD, the prevalence of hypertension, beta-blocker use, and NT-proBNP levels increased with the deterioration of renal function, but Hb levels decreased. Secondly, baseline NT-proBNP level was an independent predictive factor for all-cause death and MACEs in patients with stage 3 CKD, but echocardiography parameters weakened the predictive value of NT-proBNP. In particular, the risk of all-cause death and MACEs was elevated in patients with NT-proBNP levels ≥ 411.3 pg/ml in our study, suggesting this value as the cut-off NT-proBNP level for prediction of all-cause death and MACEs in old patients with stage 3 CKD. Thirdly, the risk of all-cause death was significantly higher in patients with CKD3b than in patients with CKD3a, and the prognostic value of NT-proBNP was better in CKD3a than in CKD3b.

The CKD is increasingly being recognized as a global public health problem substantially contributing to adverse clinical outcomes. Advanced age is a well-known risk factor for CKD [1], which is often associated with anemia, hypertension, and proteinuria, factors that increase the risk of cardiovascular disease and all-cause death. The increased NT-proBNP level and prevalence of hypertension, and decreased Hb level with the deterioration of renal function observed in our study, are consistent with the findings of previous studies [3]. Zhang et al. [2] found that the prevalence of CKD in the Chinese population was 10.8%, which is lower than the prevalences of 7.7% in the USA and 4.2% in Norway, and the prevalence of CKD3 was 1.6% [3, 13]. Lin et al. [14] found that stage 3 CKD was more common in older patients than in younger patients (33.5% vs. 4.1%) in Asia. Quality of life is better in stage 3 CKD than in ESRD and disease progression and adverse outcomes, such as kidney failure, cardiovascular disease, and premature death can be prevented or delayed when treatment is initiated in the early stages of disease [15]; therefore, patients with stage 3 CKD should receive comprehensive treatment. The results of two meta-analyses showed that the risk of adverse kidney outcomes and all-cause mortality is substantially higher in patients with eGFR values of 30–44 ml/min/1.73 m^2^ compared to those with eGFR values of 45–60 ml/min/1.73 m^2^ [16, 17]. This suggests that it may be appropriate to subdivide stage 3 CKD into CKD3a and CKD3b in the updated guidelines [10].

Brain natriuretic peptide (BNP) is produced as a prehormone (proBNP) by cardiac myocytes in response to ventricular wall stretching and tension [18]. Compared with BNP, NT-proBNP has a longer half-life and is more stable in vitro, making it easier to detect. Previous studies have shown that NT-proBNP is a significant predictor of cardiovascular disease and all-cause death in various populations [19, 20]; however, these studies were performed mainly in patients with ESRD on hemodialysis [5, 6]. The present study is the first to report that the NT-proBNP level is an independent predictive factor for all-cause death and MACEs in very old (≥80 years old) patients with stage 3 CKD. These results indicate that monitoring NT-proBNP level is helpful for assessing the risk of all-cause death and MACEs, correcting risk factors, and delaying the deterioration of renal function in this patient population.

There are several possible explanations for the association between elevated NT-proBNP level and risk of all-cause death and MACEs in our study. Firstly, an elevated plasma NT-proBNP level is associated with a variety of factors including old age, anemia, and combined hypertension and coronary heart disease, which are the risk factors for all-cause death and MACEs [21]. Secondly, increased plasma NT-proBNP level may indicate more advanced renal dysfunction, and deterioration of renal function is associated with all-cause death and MACEs [22]. Thirdly, elevated levels of plasma NT-proBNP denote impaired cardiac function, including latent structural heart diseases, cardiac volume overload, and myocardial damage [23]. In this study, NT-proBNP was not an independent predictor of all-cause death and MACEs after adjusting for echocardiography parameters (LVEF, LAD, and LVMI). Both abnormal echocardiography parameters and elevated plasma NT-proBNP level denote impaired cardiac structure and function, but no published studies have compared the prognostic value of NT-proBNP and echocardiography parameters.

The prognosis based on the NT-proBNP level has not been thoroughly studied in CKD patients, and there is no standard cut-off value for NT-proBNP to predict adverse events in this population. Fu et al. [24] reported that NT-proBNP predicted death with a cut-off value of 369.5 pg/ml in non-CKD patients and a cut-off value of 2584.1 pg/ml in CKD patients. In the present study, the risks of all-cause death and MACEs were particularly increased in patients with NT-proBNP levels ≥ 411.3 pg/ml, suggesting this value as an appropriate cut-off NT-proBNP level for prediction of all-cause death and MACEs in very old patients with stage 3 CKD. The cut-off value for predicting all-cause death suggested by Fu et al. was significantly higher than that of the present study (2584.1 pg/ml vs. 411.3 pg/ml). Possible explanations for this discrepancy include differences in the percentages of patients with heart failure, AF, and anemia and levels of NT-proBNP, which were higher in the study by Fu et al. [24]. In addition, the study population of Fu et al. consisted of patients with coronary artery disease, whereas patients with coronary artery disease accounted for 79.1% of the patients in our study. It is difficult to determine an explicit cut-off value for predicting all-cause death and MACEs in patients with CKD because the range of plasma NT-proBNP values is wide, especially in the old; therefore, further study is needed to determine the best cut-off value for plasma NT-proBNP in very old patients with stage 3 CKD.

Patients with CKD are at increased risk of mortality, cardiovascular disease, and less commonly, progression to ESRD [25] and these risks are increased with deterioration of renal function [26]. In the present study, the mortality rate was significantly higher in patients with CKD3b than in those with CKD3a (70.6% vs. 55.6%, *p* = 0.037) during follow-up, but the incidence of MACEs did not significantly differ (41.0% vs. 37.3%, *p* = 0.563). This discrepancy may be due to death occurring before the occurrence of MACEs in patients with CKD3b. In addition, the prognostic value of NT-proBNP for predicting mortality and MACEs was better in CKD3a than in CKD3b. The reason for this discrepancy is not clear but may be associated with the small sample size in this study. There is a different mode of clearance of the NT-proBNP in patient with CKD3b, and many other factors that have not been discussed in this study affect the prognosis of patients with CKD3b. Further studies are needed to compare the prognostic value of NT-proBNP in CKD3a versus CKD3b.

Several limitations should be considered when interpreting our results. Firstly, the study population was relatively small; therefore, large studies are needed to confirm our results. Secondly, in this patient population, numerous factors can affect plasma NT-proBNP levels and patient outcomes. Although the results were adjusted for multiple covariates that may be associated with circulating NT-proBNP levels and outcomes, there is a possibility of residual confounding factors. Thirdly, in this study, an inclusion criterion was eGFR < 60 ml/min/1.73 m^2^ calculated by the MDRD (Modification of Diet in Renal Disease) formula, which may be less representative of actual eGFR in very old patients because of reduced muscle mass [27].

## Conclusion

The NT-proBNP level is a strong and independent prognostic factor for all-cause death and MACEs in very old patients with CKD stage 3. Furthermore, patients with a NT-proBNP level ≥ 411.3 pg/ml have a significantly higher risk of all-cause death and MACEs, suggesting the potential usefulness of this NT-proBNP level as a cut-off value for predicting adverse events in this population. In addition, the prognostic value of NT-proBNP was better in CKD3a than in CKD3b.
